# The neural correlates of risky decision making across short and long runs

**DOI:** 10.1038/srep15831

**Published:** 2015-10-30

**Authors:** Li-Lin Rao, John C. Dunn, Yuan Zhou, Shu Li

**Affiliations:** 1 Key Laboratory of Behavioral Science and Magnetic Resonance Imaging Research Center, Institute of Psychology, Chinese Academy of Sciences, Beijing 100101, China; 2 School of Psychology, University of Adelaide, Australia

## Abstract

People frequently change their preferences for options of gambles which they play once compared to those they play multiple times. In general, preferences for repeated play gambles are more consistent with the expected values of the options. According to the one-process view, the change in preference is due to a change in the structure of the gamble that is relevant to decision making. According to the two-process view, the change is attributable to a shift in the decision making strategy that is used. To adjudicate between these two theories, we asked participants to choose between gambles played once or 100 times, and to choose between them based on their expected value. Consistent with the two-process theory, we found a set of brain regions that were sensitive to the extent of behavioral change between single and aggregated play and also showed significant (de)activation in the expected value choice task. These results support the view that people change their decision making strategies for risky choice considered once or multiple times.

Decision making under risk involves the comparison of probabilistic outcomes, for example, whether it is preferable to take public transport or a taxi across town to a meeting. While public transport is reliable, you know it will get you to the meeting a little late. A taxi may get you there on time but runs the risk of being caught in traffic making you unacceptably late. Which do you choose? Researchers have formalized this problem in terms of choices between *gambles*. A gamble is expressed as a set of outcomes and associated probabilities, e.g., a 50% chance of gaining $200 coupled with a 50% chance of losing $100. As in the above example, people are sometimes asked to choose between a gamble and *certainty* – a special kind of gamble in which there is only one outcome (occurring with 100% chance).

Choice between gambles is thought to be based on comparison of the overall value or utility of each gamble. One possible valuation function is that which maximizes the overall objective payoff. This function can be shown to correspond to the *expected value* of the gamble. Given a set of payoffs, *x*_1_, *x*_2_, …, *x*_*n*_, and a set of associated probabilities, *p*_1_, *p*_2_, …, *p*_*n*_, the expected value (EV) of the gamble is the sum, 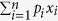. Despite its simplicity, or perhaps because of it, it is easy to show that people do not choose between gambles in accordance with their expected values. This has led to two main lines of response. The first is to generalize the concept of expectation. Thus, von Neumann and Morgenstern^1^ developed utility theory by replacing each objective value, *x*, by a corresponding personal utility, conceptualized as a monotonic, concave function, *u*(*x*). Similarly, Savage^2^ proposed a further generalization by replacing objective probabilities, *p*, by subjective probabilities, reflecting a person’s belief in the likelihood of an outcome. More recently still, Kahneman and Tversky^3^ proposed that probabilities be replaced by a weighting function, *π*(*p*), the products of which are not true probabilities, and that subjective utility be judged from a personal reference point which may not be a true zero-point. At each step, although the underlying theory has become more complicated, it still can be shown not to account for all the patterns of choice made by the majority of people^4^. This has led to the second kind of response in which the fundamental idea of expectation is abandoned. In this approach, it is assumed that choices between gambles are based on a comparison of their component features. For example, the *priority heuristic* proposed by Brandstätter, *et al.*^4^, assumes that people choose between two gambles by comparing, in sequence, the minimum outcome (or payoff) of each, the probability of the minimum outcome, and the maximum outcome, until a clear difference is obtained. Similarly motivated accounts have been proposed by Li^5^ and Thorngate^6^. A characteristic feature of this approach is that decision rules tend to be simple and based on a principle of *satisficing*^7^ rather than optimizing.

Although in many instances, people do not base their choice between gambles on the expected values, their overall propensity to do so increases if they are asked to consider the same gamble as an *aggregated* gamble in which the simple gamble is played repeatedly. This was first (or most famously) considered by Samuelson^8^ who noted that an unnamed colleague, who had declined the offer of a gamble consisting of a 50% chance of gaining $200 and a 50% chance of losing $100 (in contrast to the certainty of zero change), was prepared to accept the gamble if he was permitted to play it 100 times. The gamble in question has a positive EV, whether played once or repeatedly, and so should in each case be preferred to the certain outcome. In the eyes of Samuelson’s colleague, the positive EV became more attractive when aggregated over repeated plays.

Subsequent research has confirmed an increased tendency for people to choose the gamble with greater EV under repeated play e.g.,^9^,^10^,^11^,^12^,^13^,^14^,^15^. The same effect is observed when the EV is both positive and negative, and when the repeated plays are either *segregated* or aggregated^16^. A gamble is segregated over *n* plays when the payoffs are reduced by a factor of *n* on each play resulting in the same EV for both single and repeated plays. For Samuelson’s gamble, if it is segregated over 100 plays then each play consists of a 50% chance of gaining $2 and a 50% chance of losing $1. As discussed more fully below, on many criteria, a segregated gamble is more attractive than its single-play counterpart. However, this is not the case for an aggregated gamble although people show similar EV-consistent preferences for both.

## The actual and perceived structure of repeated gambles

**Figure 1:**
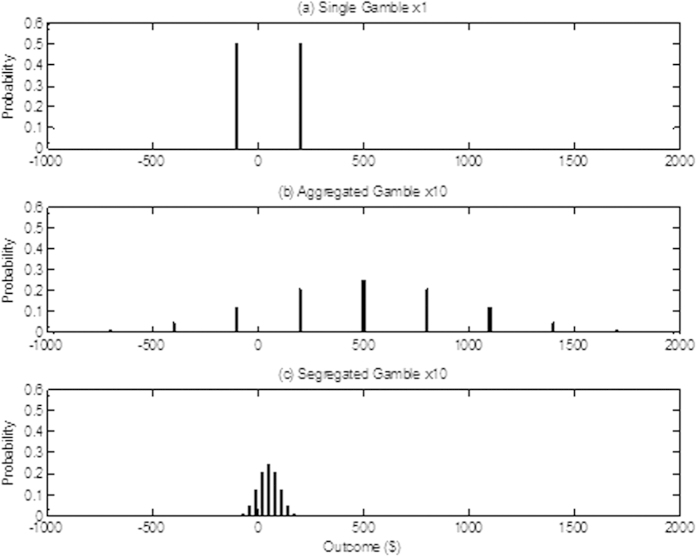
Comparison of the structure of the Samuelson gamble played in three different ways; (**a**) played once as a single gamble; (**b**) played 10 times as an aggregated gamble; and (**c**) play.ed 10 times as a segregated gamble. Vertical bars represent the probability of each outcome in dollars.

A gamble, such as the one offered to Samuelson’s colleague, changes its payoff structure if played more than once^17^. Figure 1 shows the structure of the Samuelson gamble played once (single gamble), played 10 times (aggregated gamble), and played 10 times at 1/10 of the payoff (segregated gamble). In single play, there are only two possible outcomes (win $200 or lose $100), while if played 10 times, there are 11 possible outcomes. In general, if a binary gamble (one with two options) is played *n* times, there are *n* + 1 possible outcomes with probabilities that follow the binomial distribution. In aggregated gambles, the outcomes expand around the expected value ($500 in Fig. 1b) while in segregated gambles they contract around the expected value ($50 in Fig. 1c). As a result, many features of a gamble also change from single to repeated play. In an aggregated gamble, the EV increases with *n*, as do all gains and losses, while in a segregated gamble, all these values remain constant. The standard deviation of payoffs increases for aggregated gambles and decreases for segregated gambles and, consequently, the probability that an outcome will fall in a specified interval centered on the EV (the *probability interval* or PI) decreases for aggregated gambles and increases for segregated gambles. For both aggregated and segregated gambles, the probability of a loss decreases with *n* but the associated *mean excess loss* (the average amount lost given that a loss is made) decreases for segregated gambles and increases for aggregated gambles (in the form of a non-monotonic saw tooth function). It can readily be shown that for an aggregated gamble, 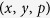, played *n* times, the expected value is 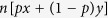 and the variance is 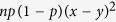. For the same segregated gamble played *n* times, the expected value is 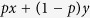 and the variance is 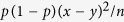.

While it is an open question if people are able to compute the distributions corresponding to aggregated and segregated gambles without formal mathematical training, they are nevertheless sensitive to changes in various features of these gambles as a function of *n*^18^. In their study, Klos *et al.* asked participants to estimate four objective features of four different gambles (one of which was the Samuelson gamble shown in Fig. 1) each of which could be played once, 5 times, or 50 times. Thus, the repeated plays corresponded to an aggregated gamble. The four objective features were probability of a loss, mean excess loss, the standard deviation of outcomes, and the PI. For the first three features, participants correctly identified the direction of change with increasing *n* although they tended to overestimate their values, at least for 50 plays. Yet, despite the fact that they correctly believed the standard deviation increases with *n*, people also reported incorrectly that the PI *decreases* with *n*. Consistent with this, their perceived risk decreased and their acceptance rates increased with *n* (similar to Samuelson’s colleague).

## Theories of single-repeated play difference

The increased propensity to choose the gamble with greater EV when played repeatedly (either as a segregated or aggregated gamble) poses a challenge to most theories of risky decision making. For example, according to the priority heuristic proposed by Brandstätter, *et al.*^4^, in a mixed gamble involving both gains and losses, people first compare options in terms of their minimum outcomes. However, in a single play version of the Samuelson gamble, the minimum outcomes are -$100 (if the gamble is accepted and lost) and $0 (if the gamble is declined). The difference between these values is then compared to the aspiration level corresponding to one tenth of the highest absolute gain or loss ($200). Since the difference of 100 is greater than the aspiration level of 20, the priority correctly predicts that Samuelson’s colleague will refuse the single play gamble. But for a repeated gamble aggregated *n* times, the difference in minimum gains is 100*n* and the aspiration level is 20*n*, leading to the same decision. The priority heuristic therefore cannot account Samuelson’s colleague greater preference for the aggregated gamble.

Expectation theories are also challenged by the shift in preferences from single to repeated play. The challenge is of two kinds. First, in order to maximize an expectation in the repeated case, participants are required to “unpack” the text and recover the implied probability distributions as illustrated in Fig. 1. It is unclear if such a complex process is psychologically plausible (at least without mathematical training). Second, even if this process is assumed, a very specific form of the utility function must be chosen in order to account for the observed effects^19^,^20^.

Although the shift in preference for single and repeated plays of a gamble poses difficulties for existing theories, we choose to focus on a more fundamental question. This is whether people base their decisions on the same process or strategy mix under single and repeated play or if they shift their decision making strategies from single to repeated play. While theories of risky choice implicitly suppose that people all follow the same choice strategy (whatever that is), it seems more likely that different people may employ different strategies and that the same person may use different strategies for different choice problems. We assume that different people, or the same person at different times, use a mix of different strategies to choose between gambles. Some strategies may be based on maximizing a valuation function (such as EV) while others may be based on a comparison of features (such as the priority heuristic).

Given that people may use a mix of strategies, the shift in preferences to EV consistent choice from single-play to repeated-play may arise for one of two reasons. First, if people use the same mix of strategies in single and repeated play, and these strategies are sensitive to the change in the structure of the gamble, then this may result in an increased preference for the option with greater EV. We call this the *one-process theory*, because the same process, or set of strategies, is used in both single and repeated play. As noted earlier, existing one-process theories (such as the priority heuristic or prospect theory) are unable to account for the shift in preferences between single and repeated play. However, because different features of the gamble change (or appear to change) between these two kinds of play, to the extent that they are relevant to people’s decision making, the nature of their decisions may also change. For example, Klos *et al.*^18^ found that people believe that the probability interval is less for repeated play than for single play and so, if this feature is relevant to their decision making in the same way for both kinds of gamble, it follows that different values may lead to different choices. It is beyond the scope of the present paper to develop a specific account of this type since any of a large number of features, or combinations or features, may be relevant. For this reason, we aim to test all possible single-process accounts simultaneously.

The single-process theory assumes that the same strategies (or sets of strategies) are used in both single and repeated play. In contrast, the *two-process theory* proposes that people change their strategy mix from single to repeated play, specifically by adopting an EV maximization strategy in repeated play. We call this the two-process theory because different processes, or strategies, are used between single and repeated play.

One way of distinguishing between the one-process and two-process theories is by means of functional MRI. Our logic is as follows. If the one-process theory is correct then the principal difference between single and repeated play arises from the calculation and evaluation of relevant features of the gamble. In contrast, if the two-process theory is correct then the principle difference between single and repeated gambles arises from the different strategies used which will include calculation and evaluation of EV (or other statistics that depend upon this calculation). In order to identify the neural correlates of EV calculation, as well as asking participants to choose between gambles and certain outcomes under single and repeated play, we also asked them to choose on the basis of EV. If the two-process theory is correct then brain regions that are sensitive to the difference in preferences between single and repeated play should also show enhanced sensitivity to preferences explicitly based on EV. In contrast, if the one-process theory is correct then brain regions that are sensitive to the difference in preferences between single and repeated play should be unaffected by preferences explicitly based on EV.

In order to identify brain regions sensitive to a shift in preference from single to repeated plays, we presented participants with two kinds of gamble; a low risk/low payoff (LL) gamble, and a high-risk/high payoff gamble (HH), each of which is compared to a certain outcome. The LL gamble is similar to the “Bet P” gamble studied by Lichtenstein and Slovic^21^ and offers a high probability (80%) of winning a modest amount of money (either Ұ30, Ұ33, Ұ37, or Ұ40) and a low probability (20%) of losing an even more modest amount of money (Ұ-20). We constructed LL gambles in which the EV was always greater than the associated certain outcome of between Ұ16 and Ұ20. Despite its greater EV, the risky choice in the LL gamble tends to be relatively unattractive due to the *certainty effect* whereby people prefer a certain outcome over a risky one of equal or greater value^3^. The HH gamble is similar to the “Bet $” gamble studied by Lichtenstein and Slovic^21^. It offers a low probability (20%) of winning a relatively large amount of money (Ұ70, Ұ73, Ұ77, and Ұ80) and a high probability (80%) of losing a small amount of money (-Ұ1). We constructed HH gambles in which the EV was always less than the associated certain outcome (also between Ұ16 and Ұ20). Despite its lower EV, the risky choice in the HH gamble tends to be relatively attractive due to the *possibility effect* whereby people prefer a risky outcome with a high payoff as long as it is merely possible^3^.

The combination of LL and HH gambles allows us to distinguish a greater preference for the EV consistent choice from an overall increase in risk propensity in aggregated gambles. This is because greater preference for the EV maximizing choice corresponds to an *increased* preference for the risky option in LL gambles and *decreased* preference for the risky option in HH gambles. We use this pattern of choice as a marker to identify brain regions associated with the shift to greater EV preference.

## Method

### Participants

Twenty-seven undergraduate and graduate students (15 males, mean age 23.63 years, SD 2.13) with normal or corrected to normal vision participated in this study. Six participants were excluded because of excessive head motion (>3 mm) leaving a total of 21 participants whose data were analyzed. All participants were in good health with no previous history of psychiatric or neurological disease, and each gave written informed consent.

### Ethics statement

The study was approved by the Institutional Review Board of the Institute of Psychology, the Chinese Academy of Sciences, and the Institutional Review Board of the Beijing MRI Center for Brain Research. The methods were carried out in accordance with the approved guidelines.

### Procedure

**Figure 2:**
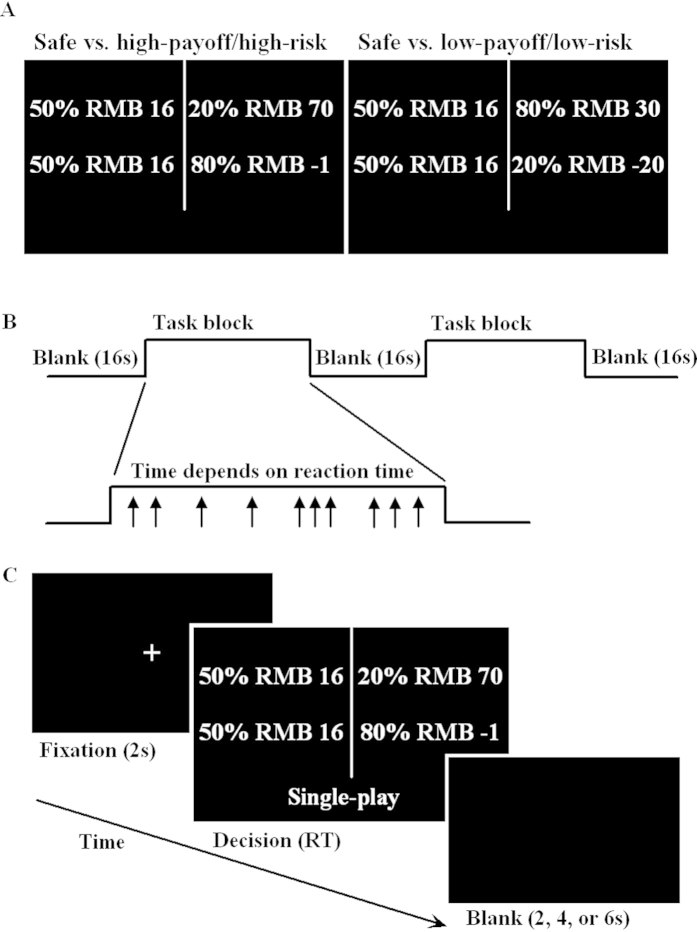
Diagram of the stimulation paradigms : (**A**) Trial types. (**B**) Schematic of the mixed design. (**C**) Trial timing. Each paradigm is illustrated with respect to the single gamble.

Each participant was presented with 40 gambles each consisting of a pair of options, one of which was certain, the other risky (see Fig. 2). The values of the certain options varied from Ұ16 to Ұ20 in integer increments. Half the risky options were LL gambles, the other half were HH gambles.

Participants responded to each gamble under three different instructions or tasks. In the single gamble task, participants were asked to choose between the two options under the assumption that their selection would be applied only once. In the aggregated gamble task, participants were asked to choose between the two options, under the assumption that their selection would be applied a total of 100 times. In the EV judgement task, participants were asked to select the option with the greater expected value for each gamble. Prior to commencing this task, participants were told how to calculate an expected value.

Participants performed each task in the fMRI scanner. Prior to entering the scanner, each participant played a practice version of each task to minimize learning effects during the actual scanning and to enable them to fully understand the paradigm. The options used in the practice phase were unrelated to those used in the test phase. During scanning, the participants were allowed to perform the tasks with no time constraints. The participants’ choices and response times were recorded.

The trials were blocked by task, with 10 trials per block and 12 blocks presented in a pseudorandom order, which ensured that each of the three tasks was presented twice within every set of 6 blocks in each of two runs. A block consists of a 6 sec presentation of the task instruction, followed by 10 test trials. On each trial, participants were presented with a pair of options and asked to make their decision by pressing one of two buttons corresponding to the location of the options on the screen. The presentation of gambles was counter-balanced across left and right sides of the screen. After a participant’s response, there was a variable delay (2, 4, or 6 sec) before presentation of a fixation cross indicated that the next trial would begin in 2 sec (see Fig. 2). At the end of each block of trials, there was a 16 sec blank interval before the next block began.

The participants were informed that they would be paid Ұ100 for session. To further incentivize their cooperation, they were also told that at the end of the experiment that one single-play and one aggregated-play choice would be randomly selected to be played in actuality (either as a single-play or as an aggregated-play), with the relevant outcomes determined at random by a Matlab® program. For the single-play choice, the selected choice would be played only once. For the aggregated-play choice, the selected choice would be played for a total of 100 times. If the result turned out to be a gain, the participants would receive that amount in addition to their Ұ100 payment for the session. If the result turned out to be a loss, this amount was deducted from their Ұ100 payment.

### fMRI acquisition

MR images sensitized to changes in BOLD signal levels were obtained by an echo planar imaging sequence on a 3.0-Tesla Siemens MR scanner (repetition time = 2000 msec; echo time = 30 msec; flip angle = 90^o^, matrix = 64 × 64; field of view = 220 × 220 mm^2^; slice thickness = 3 mm; slice gap = 1 mm). Each brain volume was composed of 32 axial sections. Stimuli were presented with E-prime software (Psychology Software Tools, Pittsburgh, PA) on a personal computer, back-projected onto a screen using a liquid crystal display projector and viewed by the participants through a mirror mounted on the MRI head coil.

### fMRI data preprocessing

Data preprocessing and analyses were conducted with SPM8 software (Wellcome Department of Cognitive Neurology, University College London, London, UK, http://www.fil.ion.ucl.ac.uk/spm) running under Matlab 7.10 (The MathWorks, Inc, Natick, Massachusetts). Functional images were slice time-corrected to the onset of the middle slice and spatially realigned using a six-parameter affine transformation. Based on a visual inspection of the motion correction estimates, six participants who had more than 3-mm maximum displacement in any of the x, y or z directions or more than 3° of angular rotation about any axis were excluded from this study. The realigned images were spatially normalized to the standard EPI template, resampled to 3mm × 3mm × 3mm and subsequently smoothed with a Gaussian kernel of 8 mm full-width at half-maximum. Motion parameters were stored and used as nuisance variables in the following analysis. A high-pass filter with a cutoff period of 128s was used to remove low-frequency noise.

### fMRI data analyses and statistics

Event-related analysis was performed using a general linear model (GLM). Events were modeled at the time of option presentation with a duration equal to the reaction time for that trial. To identify brain regions showing a difference between single-play and aggregated-play choices, contrast maps were calculated for the aggregated-play task versus the single-play task for each participant in the first-level analysis. Second-level random effect analysis was performed with individual the ΔEV index as a covariate (see below). To further examine whether brain regions identified in the t-test analysis were involved in performing EV calculation, individual contrast maps were generated for the EV judgment task compared to the baseline in the first-level analysis. Second-level random effect analysis was performed with brain regions identified in the t-test analysis as a mask. The average values of effect size in regions showing a significant effect were extracted from the individual subject images for post hoc analyses. Significant activations of interest were identified with voxelwise *p* < 0.005 in conjunction with clusterwise *p* < 0.05 for multiple comparisons correction at the cluster level (family-wise error [FWE]).

## Results

### Risky choice

Table 1 shows the mean proportion of risky choice in each condition. These data were analyzed by a 3 (Task: single-play, aggregated-play, EV judgment) × 2 (Gamble Type: HH, LL) ANOVA. The effect of Gamble Type was significant, *F*(1,20) = 28.44, *p* < 0.001, and there was a significant Gamble Type x Task interaction, *F*(2,40) = 39.40, *p* < 0.001. As expected, the proportion of risky choices from single-play to aggregated-pay to EV-judgment decreased for HH gambles and increased for LL gambles. Apart from a few small variations, all participants successfully selected the EV-consistent choice in the EV judgment task (almost 0% risky choice for HH gambles, and almost 100% risky choice for LL gambles). Simple effect analysis revealed that for HH gambles, the proportion of risky choice in the EV judgment task was significantly less than in both the single-play and the aggregated-play tasks (*p*s ≤ 0.001), although the difference between the latter two tasks was not significant. For LL gambles, the proportion of risky choice was significantly greater in the EV judgment than in both the single-play and the aggregated-play tasks (*p*s < 0.001), and the difference between these tasks was also significant (*p* = 0.001).

**Figure 3:**
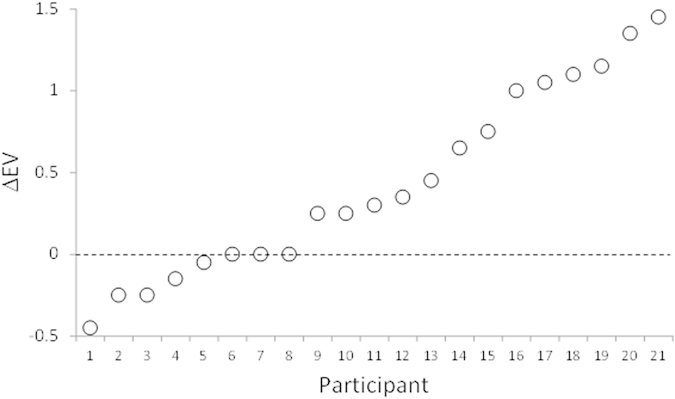
The distribution of ΔEV across participants. Higher values of ΔEV indicate a greater tendency to choose the option with greater EV in the aggregated-play task compared to the single-play task.

We created an index, called ΔEV index, that measured the extent to which each participant’s choice was more EV-consistent in aggregated-play than in single-play. It was defined as an interaction contrast – the sum of the differences in EV consistent choices between aggregated and single play for LL and HH gambles. Positive values indicate a tendency to select the EV consistent option in aggregated play while negative values indicate a tendency to select the EV inconsistent option in aggregated play. Figure 3 shows the distribution of the ΔEV index for each participant ordered from smallest to largest.

### Reaction time

Mean reaction times for each condition are shown in Table 1. These data were analyzed by a 3 (Task: single-play, aggregated-play, EV judgment) × 2 (Gamble Type: HH, LL) ANOVA. This revealed a significant effect of Task, *F*(2,40) = 3.54, *p* = 0.038, and a significant effect of Gamble Type, *F*(1,20) = 18.15, *p* < 0.001. Post hoc analyses revealed that mean reaction time for the LL gamble, *M* = 4.76 sec, *SE* = 0.29 sec, was significantly greater than mean reaction time for the HH gamble, *M* = 4.07 sec, *SE* = 0.29 sec (*p* < 0.05), and participants took less time to make a single-play choice, *M* = 3.92 sec, *SE* = 0.24 sec, than to make both either an aggregated-play choice, *M* = 4.48 sec, *SE* = 0.33 sec (*p* < 0.05), or an EV judgment, *M* = 4.84 sec, *SE* = 0.42 sec (*p* < 0.05). The difference in reaction time between the latter two tasks was not significant. The difference in reaction time between single-play and aggregated-play was significantly correlated with the ΔEV index (*r* = 0.64, *p* < 0.01).

### Neuroimaging results

**Figure 4:**
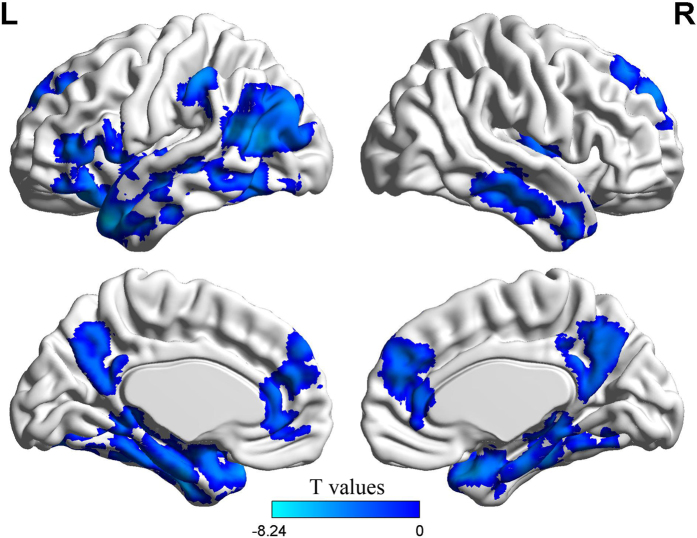
Brain regions showing a negative correlation with the ΔEV index.

**Figure 5:**
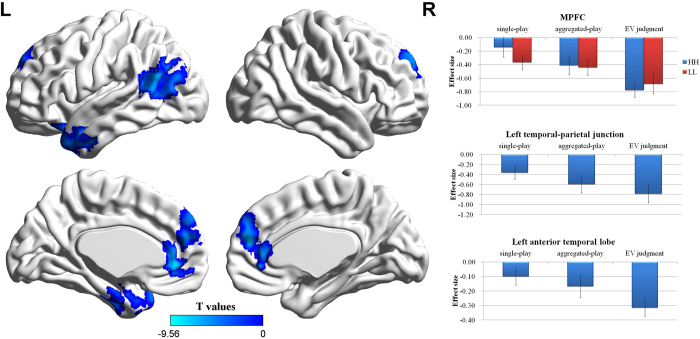
Brain regions that show both a negative correlation with the ΔEV index and significant activation/deactivation in the EV judgment task. MPFC activation (peak MNI coordinate: [3, 57, 18], corresponding *t* score = −9.56); Left temporal-parietal junction activation (peak MNI coordinate: [−51, −78, 18], corresponding *t* score = −8.42); Left anterior temporal lobe (peak MNI coordinate: [−51, 0, −24], corresponding *t* score = −6.31). Bars indicate the amplitude of the effect size in each condition or in each task. Error bar denotes the standard error.

The first analysis compared activation levels between single-play and aggregated-play tasks. No brain regions revealed a significant difference. Because our participants’ behavior showed a large individual difference (Fig. 3) and we expected that such differences would be conditional on the behavioral outcomes, we searched for brain regions in which the contrast between single and aggregated-play was significantly correlated with the ΔEV index across participants. Significant (negative) correlations were found for the bilateral superior and middle temporal gyrus, left inferior frontal gyrus, left inferior parietal lobule, right insula, anterior and posterior cingulate cortex and medial prefrontal cortex (MPFC), *p* < 0.05 (corrected). The results of this analysis are shown in Table 2 and Fig. 4. Using these brain regions as a mask, we measured the mean activation levels for the EV judgment task. We found that the temporal-parietal junction, anterior temporal lobe and MPFC each showed a significant deactivation in this task (*p* < 0.05, corrected). The results of this analysis are shown in Table 3 and Fig. 5. We then conducted a 3 (Task) by 2 (Gamble Type) ANOVA on the average value of the effect size across these three regions, with the ΔEV index as a covariate. The results revealed a significant main effect of Task for all three regions (*p*s < 0.05), and a significant interaction for MPFC (*p* < 0.05). Post hoc analyses revealed that the levels of deactivation of temporal-parietal junction and MPFC in the single-play choice were less than that in the aggregated-play choice, and that the levels of deactivation of these two regions in the aggregated -play choice were less than that in the EV judgment task (Fig. 5). The level of deactivation of the anterior temporal lobe in the EV judgment task was greater than that in the single-play choice and that in the aggregated-play choice, with no significant difference between single-play and aggregated-play choices. No other effect was found.

## Discussion

Consistent with previous research, we found that, on average, participants showed a greater preference for the option with greater expected value when presented as an aggregated gamble than when presented as a single gamble. However, we also found considerable individual differences. As shown in Fig. 3, expressed in terms of the ΔEV index, participants demonstrated the full range of choice possibilities. While some participants showed a strong shift to the EV-consistent option in aggregated-play, other participants showed little or no change, or even a change in the opposite direction (It should be noted that some participants strongly preferred the EV-consistent option even in single-play which necessarily produced an EV-index close to zero.). Because of these individual differences, we used the ΔEV index as a covariate in our fMRI analyses.

In order to distinguish between the one-process and two-process accounts, we examined whether there is an intersection between the ΔEV-moderated brain regions and brain regions associated with EV choice. We reasoned that if the two-process theory is correct then there would be a detectable intersection of the processes (and associated brain regions) between the difference in single-play and aggregated-play tasks and the EV judgment task. On the other hand, if the one-process theory is correct then there is no reason to expect an intersection with an unrelated task, such as EV judgment.

We found that at the temporal-parietal junction, anterior temporal lobe and medial prefrontal cortex (MPFC) the changes in BOLD activation from single-play to aggregated-play were significantly (negatively) correlated with ΔEV. We also found these three regions showed a significant level of deactivation in the EV judgment task. We note that these three regions are core regions of the default network, characterized as a set of brain regions that remain active during rest periods in functional imaging experiments^22^. Considering that the default network is deactivated when tasks require focused attention or high cognitive demand, our finding suggests that the aggregated-play task is associated with greater cognitive demand than the single-play task and that this difference is moderated by ΔEV. This interpretation is further supported by the observed positive correlation between ΔEV and the difference in reaction time between aggregated and single play across participants. In other words, choices in aggregated-play task take longer and are associated with greater deactivation of brain regions related to the default network in a manner proportional to the extent that behavioral choices reflect EV-maximization.

The above pattern of results, although novel, is consistent with both the one-process and two-process theories. According to the one-process account, aggregated play demands increased cognitive effort associated with the calculation of relevant properties of the options such as the probability of a loss, etc. The more effort a participant expends, the more relevant information is retrieved, and the more likely they are to select the EV-maximizing option. According to the two-process account, aggregated play demands increased cognitive effort associated with calculation of EV or similar statistics. The more effort a participant expends, the more likely they are to identify and to select the EV-maximizing option.

However, in each of the three identified regions, the level of deactivation in the EV judgment task is significantly greater than the levels of deactivation found in both single-play and aggregated-play tasks. Consistent with the two-process view, this suggests increased involvement of these regions in both the aggregated-play task (compared to single-play task) and the EV judgment task. As these three regions are core regions of the default network, the greater deactivation in the aggregated-play and the EV judgment task implies that the involvement of common processes.

The present results are consistent with those found by Su, *et al.*^23^. This study also compared risky choice in single-play and aggregated-play, and compared these to an EV judgment task, but examined eye-movement patterns rather than changes in BOLD activation. They found that the pattern of eye-movements in the aggregated-play task was similar to the pattern of eye-movements in the EV judgment task, and both were dissimilar to the pattern observed in the single-play task. This suggests that participants were attending to the expected values of the gamble options to a greater extent in the aggregated-play task than in the single-play task and were using this information to inform their preferences.

Based on the distribution of the ΔEV index, it is unlikely that all participants used the same set of strategies for single and aggregated play. Our results suggest that the mix of strategies changes when the options are presented as an aggregated gamble than when they are presented as a single gamble. If so then this has implications for studies of decision making in behavioral economics in which participants are commonly paid in real money according to their choices. Usually, two methods are used to determine participants’ payment. One is to randomly select one of the choices made by the participant during the experiment, paying the participant the outcome of that choice e.g.,^24^. The other is to accumulate all the wins and losses with the participant paid the cumulative earnings e.g.,^25^. The difference between these two payment methods is similar to the distinction between single and aggregated plays in the present study. Our results suggest that the payment methods may interact with participants’ strategy use to produce different decisions. Specifically, the aggregated earnings method may encourage greater reliance on EV maximization compared with the selected gamble method.

In summary, we have found a set of brain regions that mediate the behaviorally-moderated difference in risky choice between single and aggregated plays. A subset of these regions also show significantly greater deactivation when participants are asked to choose between the same options based on calculation of their expected values. This pattern of results is consistent with the view that (in many cases) participants change their decision making strategies to become more EV-consistent from single to aggregated play.

## Additional Information

**How to cite this article**: Rao, L.-L. *et al.* The neural correlates of risky decision making across short and long runs. *Sci. Rep.* **5**, 15831; doi: 10.1038/srep15831 (2015).

## Figures and Tables

**Table 1:** Mean proportion of risky choice and mean reaction time for each condition (standard deviations in parentheses).

Variable	Gamble Type	Task		
		Single-play	Aggregated-play	EV judgment
Risky choice				
	HH	0.50 (0.34)	0.35 (0.38)	0.02 (0.04)
	LL	0.38 (0.34)	0.66 (0.31)	0.96 (0.11)
Reaction time				
	HH	3.59 (1.31)	4.14 (1.59)	4.48 (2.14)
	LL	4.25 (1.16)	4.82 (1.84)	5.20 (1.85)

**Table 2:** Brain regions showing a significant negative correlation with individual ΔEV index.

Region	Cluster size	Peak T values	Peak MNI coordinates
Left superior and middle temporal gyrus, left inferior frontal gyrus, left inferior parietal lobule	4255	−8.24	−45 18 −33
Right superior frontal gyrus, medial prefrontal cortex and anterior cingulate cortex	642	−5.14	15 39 51
Right insula and middle temporal gyrus	483	−4.82	42 −15 0
Posterior cingulate cortex	379	−4.22	9 −60 39

**Table 3:** Brain regions that show both a negative correlation with the ΔEV index and significant activation/deactivation in the EV judgment task.

Region	Cluster size	Peak T values	Peak MNI coordinates
MPFC	434	−9.56	3 57 18
Left temporal-parietal junction	477	−8.42	−51 −78 18
Left anterior temporal lobe	371	−6.31	−51 0 −24
